# Prognostic role of renal replacement therapy among hospitalized patients with heart failure in the Brazilian national public health system

**DOI:** 10.3389/fcvm.2023.1226481

**Published:** 2023-08-23

**Authors:** Andréa Coy-Canguçu, Lígia M. Antunes-Correa, Marilda Mazzali, Paula Abrão, Fernanda Ronco, Cinthia Montenegro Teixeira, Karynna Pimentel Viana, Guilherme Cordeiro, Mauricio Longato, Otávio Rizzi Coelho, José Roberto Matos-Souza, Wilson Nadruz, Andrei C. Sposito, Steffen E. Petersen, Michael Jerosch-Herold, Otávio Rizzi Coelho-Filho

**Affiliations:** ^1^ Catholic Pontifical University of Campinas Medical School, Campinas, Brazil; ^2^ Department of Medicine, State University of Campinas School of Medical Sciences, Campinas, Brazil; ^3^ AstraZeneca Brasil, São Paulo, Brazil; ^4^ Analytix, São Paulo, Brazil; ^5^ William Harvey Research Institute NIHR Barts Biomedical Research Centre, Queen Mary University London, London, United Kingdom; ^6^ Barts Heart Centre, St Bartholomew’s Hospital, Barts Health NHS Trust, London, United Kingdom; ^7^ Non-Invasive Cardiovascular Imaging Program, Department of Radiology, Brigham and Women’s Hospital and Harvard Medical School, Boston, MA, United States

**Keywords:** heart failure, hospitalization, mortality, renal replacement therapy, chronic kidney disease, acute kidney injury, cardio-renal syndrome

## Abstract

**Introduction:**

Data on patients hospitalized with acute heart failure in Brazil scarce.

**Methods:**

We performed a cross-sectional, retrospective, records-based study using data retrieved from a large public database of heart failure admissions to any hospital from the Brazilian National Public Health System (SUS) (SUS Hospital Information System [SIHSUS] registry) to determine the in-hospital all-cause mortality rate, in-hospital renal replacement therapy rate and its association with outcome.

**Results:**

In total, 910,128 hospitalizations due to heart failure were identified in the SIHSUS registry between April 2017 and August 2021, of which 106,383 (11.7%) resulted in in-hospital death. Renal replacement therapy (required by 8,179 non-survivors [7.7%] and 11,496 survivors [1.4%, *p* < 0.001]) was associated with a 56% increase in the risk of death in the univariate regression model (HR 1.56, 95% CI 1.52 -1.59), a more than threefold increase of the duration of hospitalization, and a 45% or greater increase of cost per day. All forms of renal replacement therapy remained independently associated with in-hospital mortality in multivariable analysis (intermittent hemodialysis: HR 1.64, 95% CI 1.60 -1.69; continuous hemodialysis: HR 1.52, 95% CI 1.42 -1.63; peritoneal dialysis: HR 1.47, 95% CI 1.20 -1.88).

**Discussion:**

The in-hospital mortality rate of 11.7% observed among patients with acute heart failure admitted to Brazilian public hospitals was alarmingly high, exceeding that of patients admitted to North American and European institutions. This is the first report to quantify the rate of renal replacement therapy in patients hospitalized with acute heart failure in Brazil.

## 1. Introduction

Despite considerable improvement in managing cardiovascular disease in recent years, hospitalization and in-hospital mortality rates among patients with acute heart failure remain high (1–8). Acute kidney injury is a common complication in these patients and is associated with an increased risk of death and high levels of resource use (9–12). The risk of acute kidney injury increases by as much as ten times in individuals with preexisting chronic kidney disease (13–15), a condition that affects over half of the population with heart failure (16) and is related to worse prognosis (17–19). The management of combined acute heart failure, acute kidney injury, and preexisting chronic kidney disease is challenging, and when edema and volume overload persist despite maximal HF therapy, renal replacement therapy may provide an alternative (20–22). However, the decision to initiate such therapy may be difficult as the appropriate timing remains uncertain (23).

There is a paucity of data available on demographic and clinical characteristics, as well as outcomes, of patients hospitalized with acute heart failure in Brazil. Despite its importance as the first and largest registry to include information on this population in different Brazilian regions, the Brazilian Registry of Heart Failure (BREATH) comprises data on only 1,261 patients over a time span of less than two years, half of them admitted in Southeast health care facilities alone (24). Furthermore, while the registry does provide mean creatinine values on admission, it does not provide data on renal replacement therapy, nor have the publications derived from the registry addressed the association between renal function and mortality (24, 25).

Therefore, we retrieved data from a large public database of patients admitted to hospitals from the Brazilian National Public Health System (SUS) [SUS Hospital Information System (SIHSUS) registry] and filtered patients hospitalized with acute heart failure between April 2017 and August 2021. The purpose of this study was fourfold: (1) to determine the in-hospital all-cause mortality rate; (2) to determine the renal replacement therapy rate and its association with in-hospital mortality; (3) to evaluate other potential risk factors for in-hospital mortality, including COVID-19 infection at admission or during hospitalization, demographic characteristics, geographic location of the hospital; and (4) to analyze the effects of therapies like renal replacement therapy, and demographic characteristics on the cost of hospitalization.

## 2. Methods

### 2.1. Study design and population

This was a cross-sectional, retrospective, records-based study performed using data extracted from the SUS Hospital Information System (SIHSUS) registry, a large database of de-identified data made publicly available by the SUS Department of Informatics (DATASUS). DATASUS is responsible for the development, implementation, and operation of the health information systems related to SUS, which is the main source of health care for 78% of the Brazilian population without private health insurance (26). All residents and visitors, including undocumented individuals, are entitled to services provided free-of-charge by SUS. Although access to information and communication technologies is still limited in certain remote locations and indigenous communities in Brazil, it is estimated that 91% of all Brazilian medical facilities with over 50 beds had electronic health record systems in 2019 (27). In this setting, the SUS Hospital Information System was created with the purpose of registering all health care services provided during hospitalizations covered by SUS to allow health managers to make the due payments to health facilities. Data used in the present study were extracted from the SIHSUS registry using the Research Electronic Data Capture (REDCap; https://www.project-redcap.org) tool, a secure web-based application for building and managing online databases for research purposes.

The study population consisted of adult patients (≥18 years) with a documented diagnosis of heart failure admitted to any public hospital included in the SIHSUS database between April 2017 and August 2021. These patients were identified when the International Classification of Diseases, 10th edition (ICD-10), codes I50.X encompassing heart failure were listed as reasons for admission. Consecutive hospitalizations of the same patient were not identifiable, nor was the start date of renal replacement therapy (i.e., patients receiving renal replacement therapy prior to hospitalization were analyzed along with patients started on this therapy during their hospital stay). The definition of renal replacement therapy adheres to established international guidelines (28). As per this definition, intermittent hemodialysis includes both intermittent conventional hemodialysis and sustained low-efficiency dialysis, while continuous renal replacement therapy specifically refers to continuous venovenous hemodialysis.

Follow-up duration spanned the time between hospital admission and discharge or death. Patients whose date of admission, date of discharge or death, age, sex, and hospital location were missing were excluded from the study. The current study, as well as the SIHSUS registry, conform to the Declaration of Helsinki. The Institutional Review Board of the State University of Campinas waived the requirement to obtain any informed consent from subjects, given that no sensitive patient health information was disclosed to the investigators (Of. CEP n° 117/2022).

### 2.2. Outcome

The primary outcome was in-hospital death for any cause. We also retrieved data on demographic characteristics (age, sex, race), comorbid conditions (diabetes, hypertension, dyslipidemia, atrial fibrillation, chronic kidney disease, chronic obstructive pulmonary disease, Chagas disease), length of hospital stay, medical procedures required during hospital stay (renal replacement therapy, coronary bypass surgery, heart transplant surgery), in-hospital COVID-19, and hospitalization-related costs (in total and per day). Renal replacement therapy included intermittent hemodialysis, continuous hemodialysis, and peritoneal dialysis. It should be noted that the reporting of race in the SIHSUS registry follows the racial classification standardized by the Brazilian Institute of Geography and Statistics (IBGE), which asks individuals to self-identify within one of the following categories: “branco” (white), “pardo” (multiracial), “preto” (black), “amarelo/asiático” (yellow/Asian), and “indígena” (indigenous).

### 2.3. Statistical analysis

Continuous variables are presented as median with interquartile range (IQR) and were compared between survivors and non-survivors using the Kruskal–Wallis test. Categorical variables are presented as counts and proportions (%) and were compared between groups with the use of Pearson *χ*^2^. A variable termed “COVID-19 pandemic” was created to account for heart failure admissions after the World Health Organization declared COVID-19 a pandemic in March, 2020. Crude survival was assessed and illustrated by Kaplan–Meier plots. After graphically checking the model assumption of proportional hazard, univariate analyses were conducted to evaluate the association between patients' characteristics and in-hospital death. Independent predictors of death were identified using multivariable Cox proportional hazard regression using stepwise variable selection. Cox regression results are presented as hazard ratios (HR) with 95% confidence intervals (CI). Only notifiable variables were included in the multivariable model, meaning all medical procedures for which an invoice is mandatory (e.g., renal replacement therapy, heart transplant), demographic variables (e.g., sex, age, race), COVID-19 status (infected or not infected), and admission pre/during COVID-19 pandemic. Because the reporting of comorbidities in the SIHSUS registry is optional, we did not include comorbidities in the multivariable model, as the risk of underreporting was likely high, but did provide HR for these comorbidities from univariate models. The cost per day of hospitalization was modeled with a generalized linear model (GLM) using a gamma distribution with a logarithmic link function. With this model, the exponentiated coefficient estimates for sex, age at admission, race, year of admission, and renal replacement therapy in the GLM represent cost multipliers. A GLM with gamma-distribution was also used to analyze duration of hospitalization. The effect of hospital location was analyzed on the scale of the five geopolitical regions or macro-regions of Brazil. These five regions, North, Northeast, Central-West, South, and Southeast, have been defined by the Brazilian Institute of Geography and Statistics (IBGE). The effects of regional disparities of longevity and income levels on in-hospital mortality among patients with heart failure were investigated using a Cox proportional hazards mixed effects model. The model included predictor variables previously identified first through stepwise selection, as well as the components of the Human Development Index (HDI), namely longevity and income level (ranging from 0 to 1), available for each of the 26 federal states and the district of Brasilia. To account for differences between states not captured by human development indices, the model included a random intercept by state. A *p*-value <0.05 was considered statistically significant. All analyses were performed using SPSS Statistics version 25.0 (IBM Corp., Armonk, NY, USA) and the R environment (version 4.1.2, https://www.R-project.org/).

## 3. Results

### 3.1. Study population and in-hospital mortality

**Figure 1:**
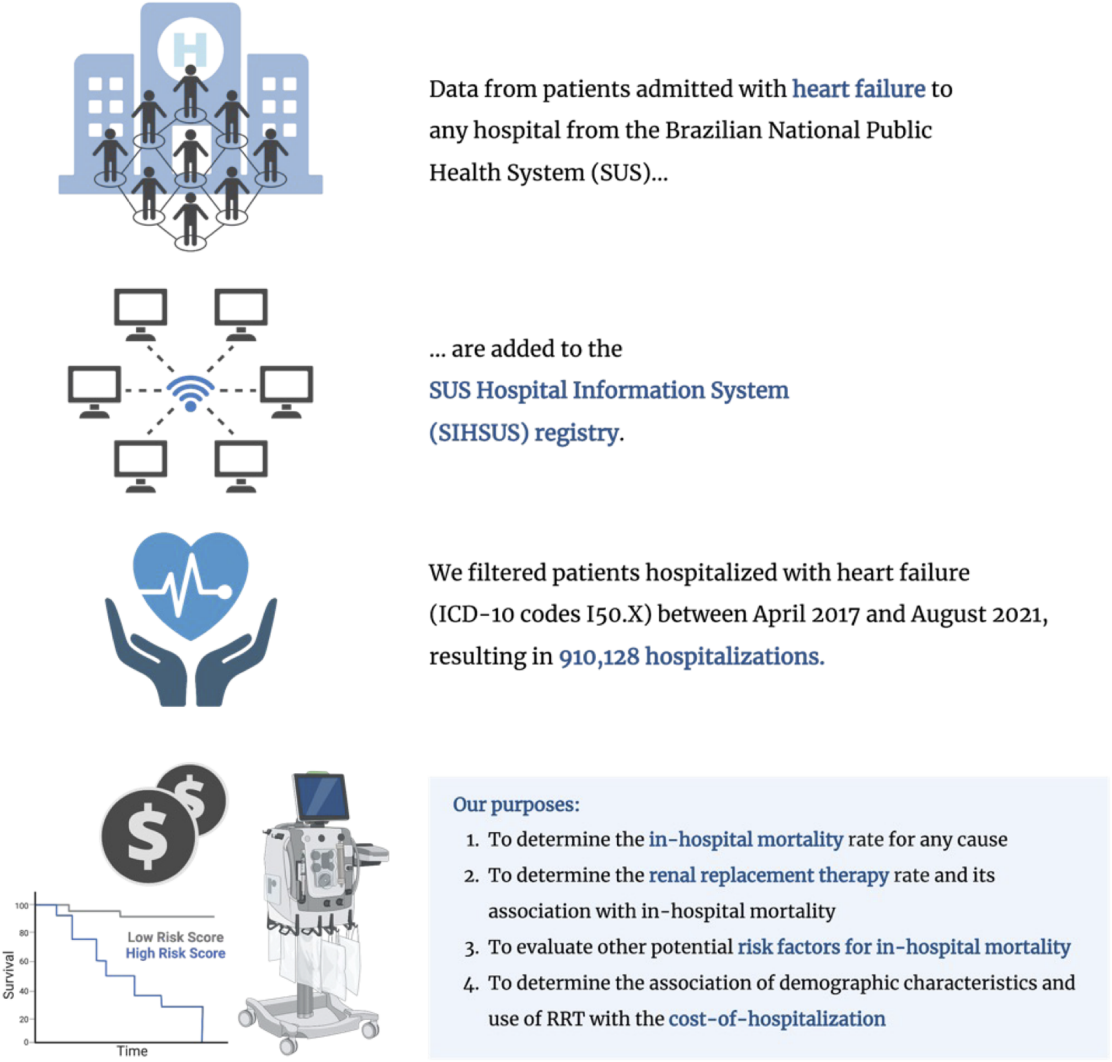
Study design. Created with BioRender.com.

In total, 910,128 hospitalizations due to heart failure between April 2017 and August 2021 were identified in the SIHSUS registry (Figure 1), of which 106,383 (11.7%) resulted in in-hospital death and 19,675 (2.2%) required renal replacement therapy. Demographic and clinical characteristics of the hospitalizations stratified by death are described in Table 1. Non-survivors, half of whom were men, had a median age of 74 years (IQR 63–83) and were mostly white (49%) and pardo (41%). Reported comorbid conditions were more prevalent in these patients compared to survivors, particularly hypertension (5.4% vs. 4.2%), chronic kidney disease (4.2% vs. 1.3%), and diabetes (2.4% vs. 1.8%, all comparisons *p* < 0.001). Survivors (48% men, *p* < 0.001) were younger (68 years, IQR 58–78, *p* < 0.001) and mainly white (49%) and pardo (42%). Median hospital stay was longer for non-survivors (Table 1). Supplementary Table S1 presents demographic and clinical characteristics based on the need for renal replacement therapy. Patients requiring renal replacement therapy during hospitalization displayed several distinctive features: younger age, higher proportion of females, longer hospital stays, increased costs, and a higher proportion requiring heart transplantation. The utilization of renal replacement therapy showed a declining trend from 2017 to 2021. Furthermore, renal replacement therapy usage exhibited non-uniform distribution across different regions of Brazil, with the southeast region having the highest prevalence among hospitalized heart failure patients.

**Table 1:** Patient demographic and clinical characteristics stratified by death.

	Survivors *N* = 803,745^a^	Non-Survivors *N* = 106,383^a^	*P*-value^b^
Age, years	68 (58, 78)	74 (63, 83)	<0.001
Female, *N* (%)	385,521 (48%)	53,456 (50%)	<0.001
Race			<0.001
White	305,530 (49%)	39,966 (49%)	
Pardo	264,361 (42%)	33,353 (41%)	
Black	40,116 (6.4%)	5,359 (6.6%)	
Asian	17,198 (2.7%)	2,047 (2.5%)	
Indigenous	762 (0.1%)	99 (0.1%)	
Length of hospital stay, days	5.0 (3.0, 9.0)	5.0 (2.0, 11.0)	<0.001
Diabetes	14,719 (1.8%)	2,536 (2.4%)	<0.001
Hypertension	34,109 (4.2%)	5,765 (5.4%)	<0.001
Dyslipidemia	321 (<0.1%)	30 (<0.1%)	0.067
Atrial fibrillation	5,340 (0.7%)	813 (0.8%)	<0.001
Stroke	1,760 (0.2%)	767 (0.7%)	<0.001
Chronic kidney disease	10,164 (1.3%)	4,431 (4.2%)	<0.001
RRT	11,496 (1.4%)	8,179 (7.7%)	<0.001
Type of RRT			<0.001
None	792,277 (99%)	98,208 (92%)	
Intermittent hemodialysis	9,707 (1.2%)	6,952 (6.5%)	
Continuous dialysis	1,559 (0.2%)	1,110 (1.0%)	
Peritoneal dialysis	202 (<0.1%)	113 (0.1%)	
COPD	7,322 (0.9%)	1,527 (1.4%)	<0.001
Chagas’ disease	811 (0.1%)	271 (0.3%)	<0.001
In-hospital COVID-19	285 (<0.1%)	100 (<0.1%)	<0.001
Coronary bypass	10 (<0.1%)	0 (0%)	0.618
Heart transplant	800 (<0.1%)	89 (<0.1%)	0.119
Hospitalization cost			
In total, R$	771 (715, 1,178)	1,020 (723, 2,602)	<0.001
Per day, R$	188 (115, 350)	354 (141, 707)	<0.001
Year of admission			<0.001
2017	186,493 (23%)	22,669 (21%)	
2018	178,553 (22%)	22,487 (21%)	
2019	177,135 (22%)	22,723 (21%)	
2020	145,981 (18%)	20,332 (19%)	
2021	115,583 (14%)	18,172 (17%)	
Geographic region of Brazil			<0.001
Central-West	57,146 (7.1%)	6,456 (6.1%)	
Northeast	181,230 (23%)	23,222 (22%)	
North	43,118 (5.4%)	5,984 (5.6%)	
Southeast	328,332 (41%)	50,026 (47%)	
South	193,917 (24%)	20,695 (19%)	

COPD, chronic obstructive pulmonary disease; RRT, renal replacement therapy, R$, currency of Brazil, the Brazilian Real [BRL, R$].

^a^ Median (IQR); *n* (%); Mean ± SD.

^b^ Kruskal–Wallis rank sum test; Pearson's Chi-squared test; Wilcoxon rank sum test; Fisher's exact test.

**Figure 2:**
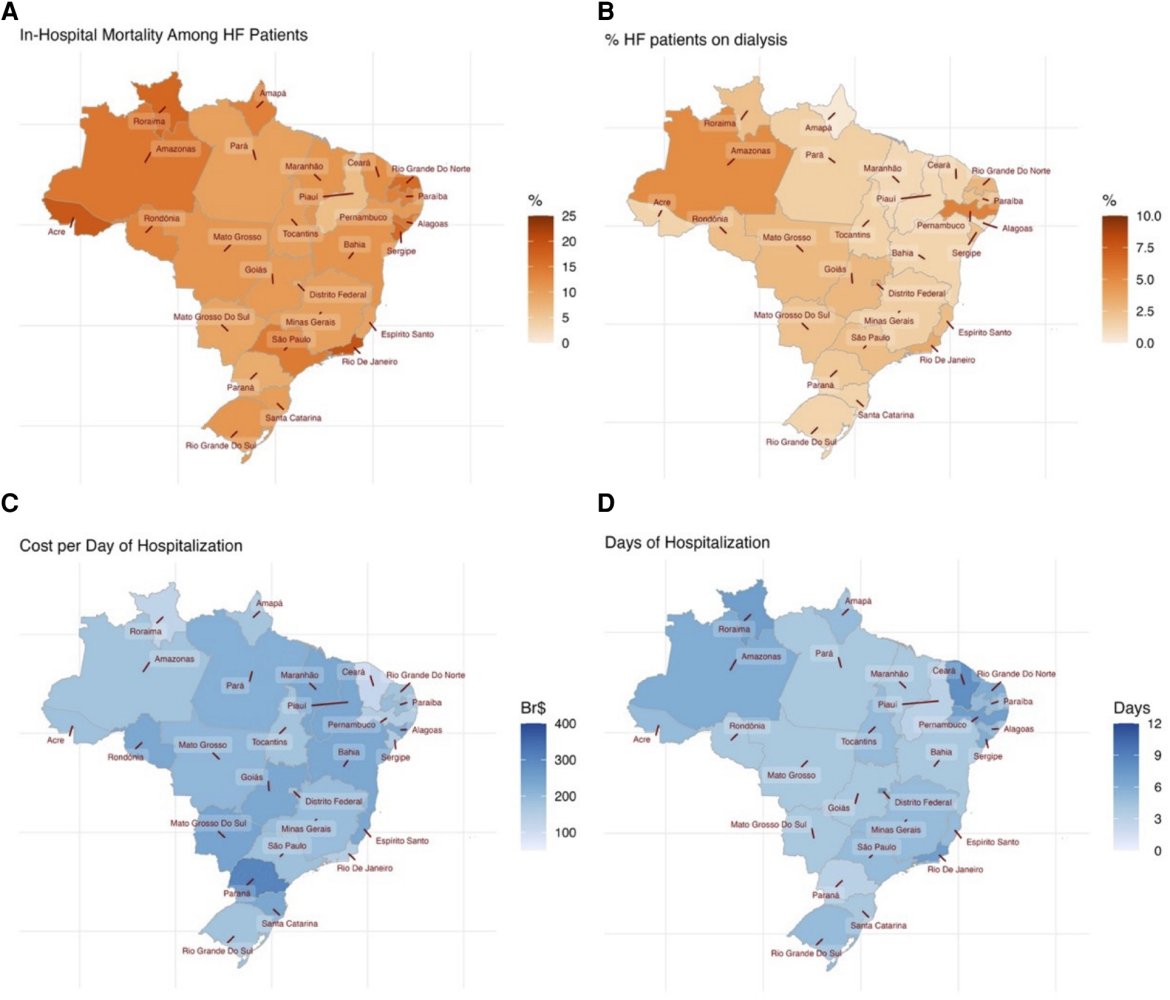
Map displays of (**A**) in-hospital mortality, (**B**) renal replacement therapy, (**C**) median cost per day of hospitalization, and (**D**) median duration of hospitalization for each federal state of Brazil.

Twelve states had an in-hospital mortality rate higher than the national rate of 11.7%, with ten located in the northern and northeastern regions of Brazil (Figure 2A). The highest rates were found in Acre (19.0%) and Rio de Janeiro (19.0%), followed by Roraima (17.0%), Sergipe (17.0%), and Rio Grande do Norte (16.0%). The lowest mortality rate was found in Piauí (6.4%), followed by Paraná (8.3%) and Distrito Federal (8.6%). Rates of renal replacement therapy were distributed relatively heterogeneously in the north and northeast of Brazil, with the highest rates in Pernambuco in the northeast (5.4%) and Amazonas in the north (5.0%) (Figure 2B), but it also included states with a renal replacement therapy rate below the national average of 2.2%: Amapá (0.3%), Alagoas (0.7%), Piauí (1.1%), Maranhão (1.1%), Bahia (1.3%), and Ceará (1.3%). All three southern states also had a renal replacement therapy rate below the national average. The states of Roraima (north) and Ceará (northeast) had the lowest median cost-per-day for hospitalization and the longest median duration of hospitalization (7 and 8 days, respectively) of heart failure patients (Figures 2C,D). The median cost-per-day was highest in the southern state of Paraná (Figure 2C) and hospitalization was a relatively short 3 days (IQR 2–6 days).

### 3.2. Renal replacement therapy and its association with in-hospital mortality

**Figure 3:**
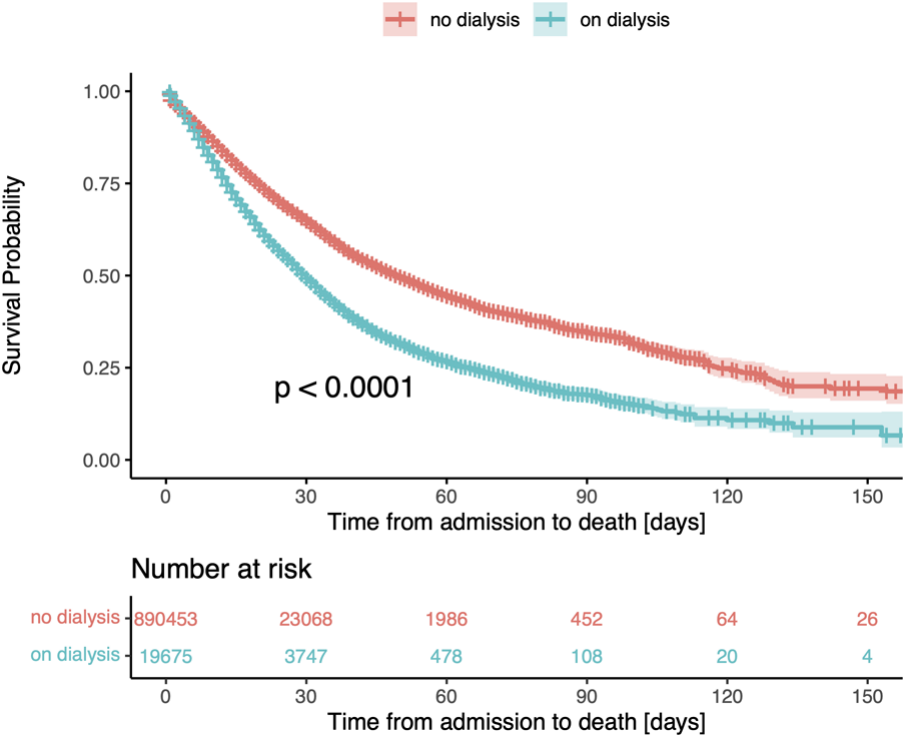
Kaplan-Meier survival curve for patients stratified by the need for renal replacement therapy (RRT). The median survival time in patients receiving renal replacement therapy was 30 days (95% CI, 29–31), compared to 50 days (95% CI, 49–51) in patients not undergoing dialysis.

Renal replacement therapy was required by 8,179 non-survivors (7.7%) and 11,496 survivors (1.4%, *p* < 0.001) (Figure 3). Intermittent hemodialysis was the most common type of renal replacement therapy employed (6.5% vs. 1.2%), followed by continuous hemodialysis (1.1% vs. 0.2%) and peritoneal dialysis (0.1% vs. <0.1%, all comparisons *p* < 0.001).

**Figure 4:**
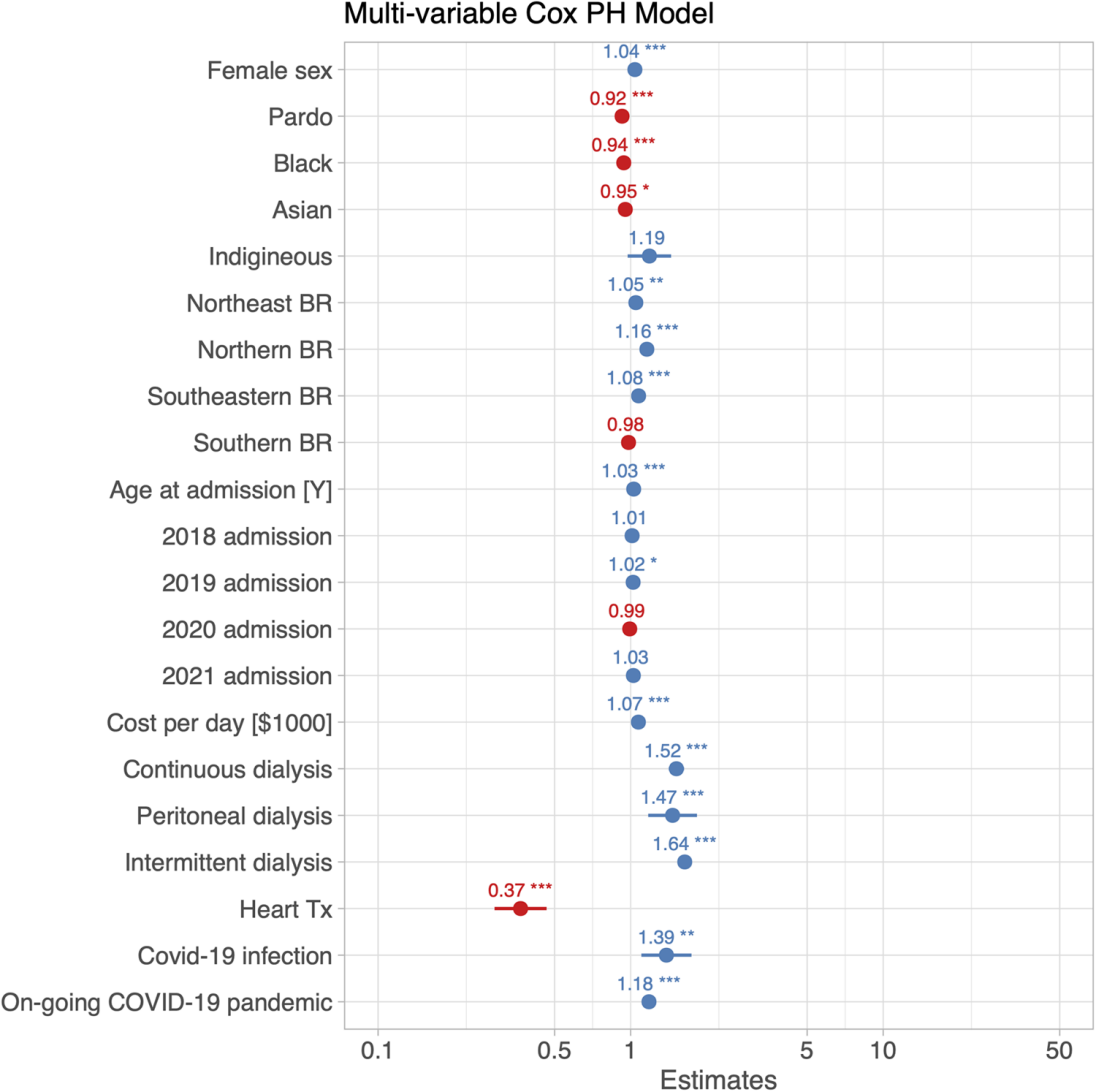
Hazard ratios with their 95% confidence intervals for the risk of in-hospital death for any cause from a multi-variable Cox proportional hazards model obtained by stepwise selection of predictors. Renal replacement therapy was associated with an approximately 56% increase of the risk of all-cause in-hospital death. The model included a variable termed “ongoing COVID-19 pandemic”, which accounted for HF admissions occurring after the official announcement of the COVID-19 pandemic by the World Health Organization (WHO) and the Brazilian Government.

In the univariate Cox regression model, the need for renal replacement therapy was associated with a 56% increase in the risk of death (HR 1.56, 95% CI, 1.52–1.59; Table 2). In the multivariable analysis (built stepwise selection of predictors), all forms of renal replacement therapy remained independently associated with in-hospital mortality (intermittent hemodialysis: HR 1.64, 95% CI, 1.60–1.69; continuous hemodialysis: HR 1.52, 95% CI, 1.42–1.63; peritoneal dialysis: HR 1.47, 95% CI, 1.20–1.88; Figure 4).

**Table 2:** Risk factors for in-hospital mortality (univariate analysis).

Description	HR	Standard error	Wald statistic	95% Confidence interval		*P*-value
				2.5%	97.5%	
Age at admission	1.027	0.0002	117.45	1.026	1.027	<0.001
Female sex	1.118	0.0061	18.25	1.105	1.132	<0.001
Race/ethnicity (reference: White)						
Pardo	0.868	0.0074	−19.12	0.855	0.880	<0.001
Black	0.859	0.0146	−10.45	0.835	0.884	<0.001
Asian	0.926	0.0227	−3.40	0.886	0.968	<0.001
Indigenous	1.090	0.1006	0.86	0.895	1.328	0.3902
Year of admission (reference: 2017)						
2018	1.017	0.0094	1.77	0.998	1.036	0.0762
2019	1.030	0.0094	3.14	1.011	1.049	0.0017
2020	1.146	0.0097	14.15	1.125	1.168	<0.001
2021	1.251	0.0100	22.51	1.227	1.276	<0.001
On-going COVID-19 pandemic	1.208	0.0066	28.78	1.192	1.223	<0.001
RRT	1.557	0.0117	37.66	1.521	1.593	<0.001
Type of RRT						
Intermittent hemodialysis	1.589	0.0126	36.68	1.550	1.629	<0.001
Continuous dialysis	1.420	0.0303	11.56	1.338	1.506	<0.001
Peritoneal dialysis	1.218	0.0942	2.09	1.013	1.465	0.0363
In-hospital COVID-19	1.626	0.1001	4.86	1.336	1.978	<0.001
Heart transplant	0.431	0.1061	−7.93	0.350	0.531	<0.001
Chagas disease	1.532	0.0608	7.01	1.359	1.726	<0.001
Diabetes	1.037	0.0211	1.799	0.997	1.079	0.072
Hypertension	1.071	0.0135	5.05	1.043	1.100	<0.001
CKD	1.504	0.0154	26.46	1.459	1.550	<0.001
Ischemic heart disease	1.417	0.0267	13.08	1.345	1.493	<0.001
COPD	1.197	0.0258	6.97	1.138	1.259	<0.001
Stroke	1.784	0.0363	15.97	1.662	1.916	<0.001
Geographic region of Brazil (reference: Central-western region, which includes Mato Grosso, Mato Grosso do Sul, and Goiás)						
Northeast	1.060	0.0141	4.17	1.032	1.090	<0.001
North	1.126	0.0179	6.62	1.087	1.167	<0.001
Southeast	1.224	0.0132	15.25	1.192	1.256	<0.001
South	1.161	0.0143	10.46	1.129	1.194	<0.001
Total hospitalization cost (log-transformed)	0.767	0.0084	−31.41	0.755	0.780	<0.001
Hospitalization cost per day (log-transformed)	6.292	0.0059	309.60	6.219	6.366	<0.001

CKD, chronic kidney disease; COPD, chronic obstructive pulmonary disease; RRT, renal replacement therapy.

### 3.3. Other risk factors for in-hospital mortality

Other parameters shown to be associated with a significant increase in the risk of death in the univariate analysis included stroke (HR 1.78, 95% CI, 1.66–1.92), Chagas disease (HR 1.53, 95% CI, 1.36–1.73), chronic kidney disease (HR 1.50, 95% CI, 1.46–1.55), ischemic heart disease (HR 1.42, 95% CI, 1.35–1.49), chronic obstructive pulmonary disease (HR 1.20, 95% CI, 1.14–1.26), and hypertension (HR 1.07, 95% CI, 1.04–1.10) (Table 2). The slight increase in the risk of death attributed to diabetes was non-significant. Because the reporting of all comorbidities is not required in the SIHSUS registry, such variables were not included in the multivariable Cox regression model due to their high probability of underreporting.

In-hospital COVID-19 and heart transplant are of notifiable nature and were therefore included in both the univariate and the multivariable models. Female sex was related to an increased in-hospital mortality in both models (univariate model: HR 1.12, 95% CI, 1.11–1.13, Table 2; multivariable model: HR 1.04, 95% CI, 1.03–1.06, Figure 4). Interestingly, pardo, black, and Asian ethnicities had a smaller risk of death compared to the white ethnic group. The risk of death was also greatest in admissions which occurred in 2020 (univariate model: HR = 1.15, 95% CI, 1.13–1.17, Table 2; multi-variable model: HR = 1.13, 95% CI, 1.11–1.12, Figure 4) and 2021 (univariate model: HR = 1.25, 95% CI, 1.23–1.28, Table 2; multi-variable model: HR = 1.21, 95% CI, 1.18–1.24, Figure 4), compared to 2017. Lastly, while a COVID-19 infection during hospitalization was linked to a greater risk of death (univariate model: HR = 1.63, 95% CI, 1.34–1.98, Table 2; multivariable model: HR = 1.41, 95% CI, 1.14–1.81, Figure 4), heart transplant was associated with improving survival (univariate model: HR = 0.43, 95% CI, 0.35–0.53, Table 2; multi-variable model: HR = 0.37, 95% CI, 0.29–0.47, Figure 4). The risk of in-hospital death increased significantly during the COVID-19 pandemic independently of whether a patient was infected or not (effect of on-going COVID-19 pandemic in univariate model: HR = 1.21, 95% CI, 1.19–1.22; multi-variable model: HR = 1.18, 95% CI, 1.14–1.23). Kaplan-Meier analysis with stratification by hospital admission before or during the COVID-19 pandemic confirmed that patients admitted during the pandemic experienced worse in-hospital survival compared to those admitted before (*p* < 0.0001, Supplementary Figure S1).

### 3.4. Duration and cost of hospitalization

**Figure 5:**
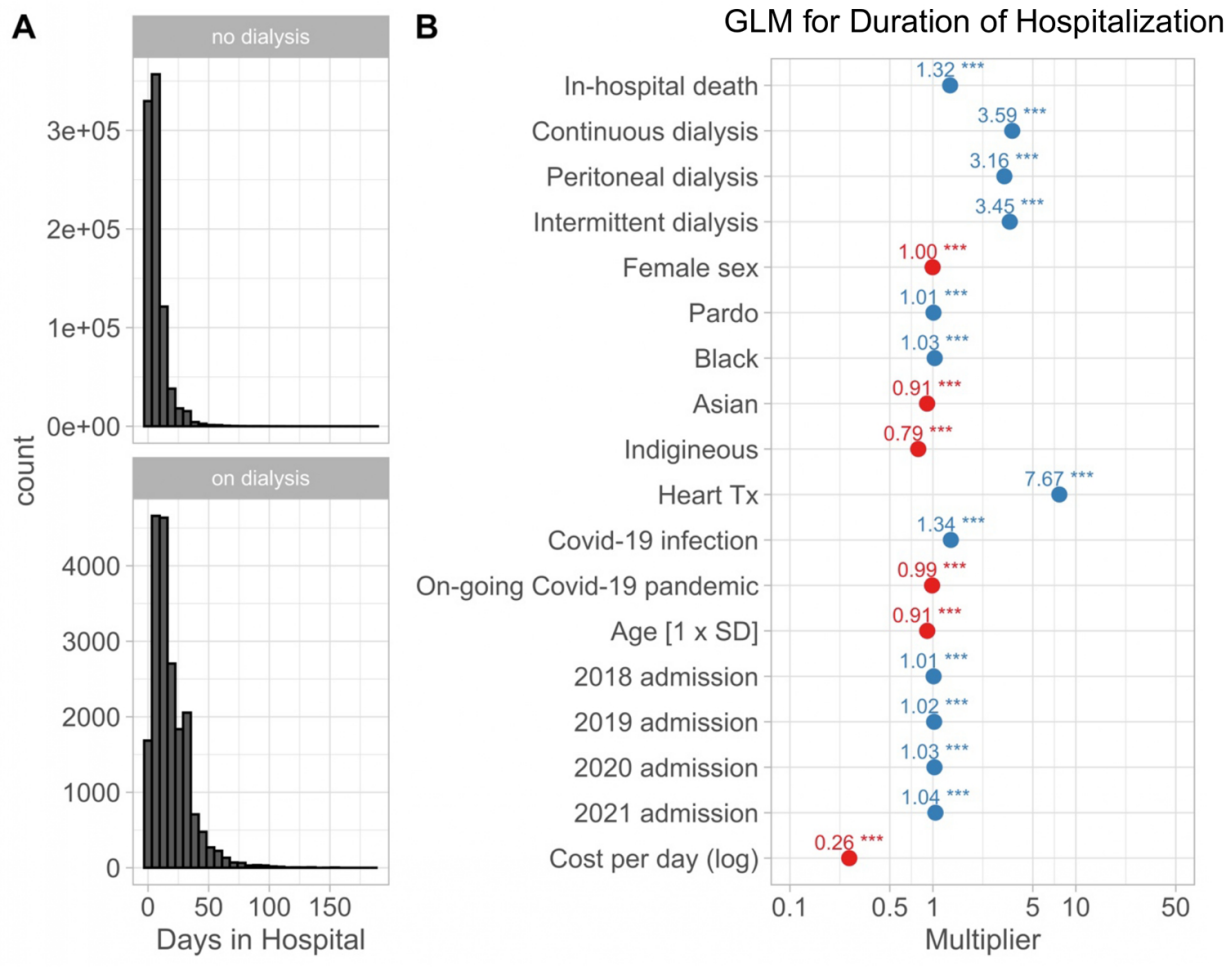
(**A**) Histograms for the duration of hospitalization in patients not receiving (top) and receiving (bottom) renal replacement therapy. (**B**) Estimated effects on the duration of hospitalization estimated with a multi-variable generalized linear model using a gamma-distribution and log-link function. The graph shows exponentiated coefficient estimates, which represent multipliers for the duration of hospitalization of 6 days for a white, male patient with age of 67 years equal to the average age for the cohort. Renal replacement therapy was associated with a more than 3-fold increase in the duration of hospitalization.

For the fourth aim of current study we found that patients requiring renal replacement therapy remained hospitalized for a longer period compared to those who did not require such therapy, as demonstrated in Figure 5A. Other factors associated with a longer hospitalization (Figure 5B) were in-hospital death, pardo or black race, heart transplantation, and in-hospital COVID-19 diagnosis. The duration of hospitalization also increased significantly from 2017, though this effect size amounted only to a 3% or smaller increase with a median duration of hospitalization of 5 days. Patients of Asian or indigenous race had shorter hospital stays, and a higher cost per day of hospitalization was also associated with a shorter hospitalization. The duration of hospitalization was shorter during the COVID-19 pandemic (*p* < 0.001), though it amounted to less than 2%.

**Figure 6:**
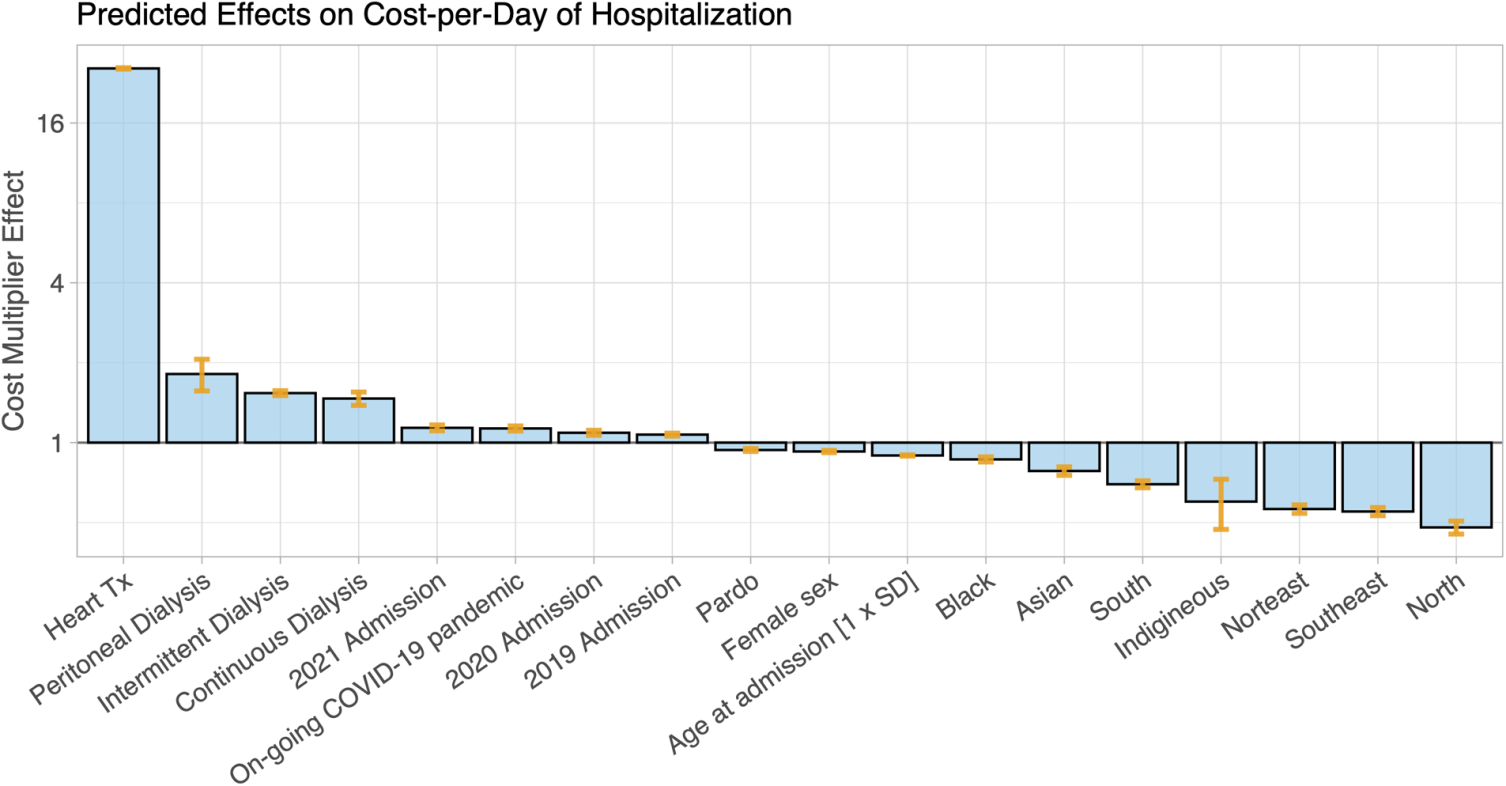
The hospitalization cost per day for patients admitted with heart failure was analyzed with a generalized linear model (GLM) with a logarithmic link in the gamma distribution function and with age, sex, ethnicity, heart-transplantation, and renal replacement therapy type included as independent predictors. The coefficients in the GLM represent cost multipliers and are shown here as bars. The estimate for the average cost of hospitalization of white female patients with a mean age of 67 years (intercept term in the model) was 603 Br$. Heart transplantation during hospitalization was by far the largest factor for cost-per-day increases, while less (cost factor <1) was spent on patients of non-white ethnicity. For the effect of geographic region, the central-western region of Brazil, which includes Mato Grosso, Mato Grosso do Sul e Goiás, was the reference region.

The median cost of hospitalizations resulting in death amounted to R$354 per day [IQR 141–707; in the currency of Brazil, the Brazilian Real (BRL, R$)], nearly twice as much as hospitalizations resulting in discharge (R$188 per day, IQR 115–350, *p* < 0.001) (Table 1). Hospitalizations during which a heart transplant or renal replacement therapy were performed had a cost-per-day approximately sixteen- and two-times greater than hospitalizations with no heart transplant or renal replacement therapy, respectively (Figure 6). In addition, the hospitalization cost-per-day continuously increased from 2018 to 2021 (Figure 6).

### 3.5. Regional disparities in in-hospital mortality among hospitalized patients with heart failure

Human development indices for longevity and income were significantly associated with in-hospital death in a multi-variate model mixed-effects Cox proportional hazards model: a one standard-deviation increase in longevity significantly decreased the risk of in-hospital death (HR = 0.80 for a 0.1 change, *p* < 0.001). Conversely, a unit increase in the income level was associated with a higher risk of in-hospital death (HR = 1.31 for a 0.1 change of the index, *p* < 0.001). Notably, these effects of longevity and income level led to a distinct north-to-south gradient of their effects (hazard ratio) on in-hospital death, but with opposite directions on the map, as displayed in the Supplementary Figure S2.

## 4. Discussion

In this large nation-wide registry of Brazilian public hospitals with 910,128 records of patients admitted with heart failure, we found an alarming mortality rate of 11.7%, and a 56% greater risk of death among patients in need of renal replacement therapy compared to patients who did not require such therapy. In addition, our findings describe female sex and COVID-19 as important risk factors for in-hospital death and highlight the prognosis-modifying effect of heart transplant in patients with heart failure in the Brazilian setting. We also report that hospitalizations requiring renal replacement therapy and hospitalizations resulting in death were both longer and costlier.

The in-hospital mortality rate of 11.7% in our study population is in line with the 12.6% rate described by the BREATH registry (24). Both rates suggest that patients hospitalized with heart failure in Brazilian health facilities have poorer survival compared to patients admitted to North American and European facilities. Data from the European Society of Cardiology Heart Failure Long-Term (ESC-HF-LT) registry, an ongoing prospective observational study comprising over 211 cardiology centers from European countries, indicate that the in-hospital all-cause mortality across the continent lies between 5.3% (29) and 5.5% (30). In the United States, death occurred in 2.9% and 5.6% of patients hospitalized with a primary and secondary diagnosis of heart failure in 2014, respectively, according to the National Inpatient Sample and the US Census Bureau (31). Notably, findings from the PARADIGM-HF trial (32) show that all-cause mortality is greatest in patients with heart failure in Latin America (HR 1.67, 95% CI, 1.28–2.19) compared to those in North America, Western Europe (HR 0.94, 95% CI, 0.75–1.17), Central/Eastern Europe and Russia (HR 1.15, 95% CI, 0.91–1.42), and Asia-Pacific regions (HR 1.40, 95% CI, 1.03–1.91).

To the best of our knowledge, no prior reports describing the rate of renal replacement therapy provision in Brazil have focused on patients hospitalized with acute heart failure. Between 2000 and 2004, 90,356 patients were started on renal replacement therapy in Brazil, corresponding to a 5.5% increase in prevalence during the study period (33). Hypertension and other cardiovascular diseases were the second prevailing cause of chronic kidney disease among these patients. Accordingly, in a prospective cohort study including 8,131 patients admitted to three intensive care units (ICU) in the Federal District of Brazil between 2017 and 2018, heart failure was reported as a comorbid condition in 12.2% and 4.9% of patients with and without acute kidney injury, respectively (34). Similar rates were also reported by Doher et al. in a retrospective cohort study of COVID-19 patients admitted to the ICU of a private quaternary hospital in São Paulo in 2020 (35). Among its thirty-four patients on renal replacement therapy due to acute kidney injury, 26.5% had a previous diagnosis of heart failure versus 4.8% of 166 non-renal replacement therapy patients.

Despite clinical practice guidelines recommending the use of renal replacement therapy to treat acute kidney injury in the setting of major metabolic and fluid disturbances (36, 37), practice patterns and outcomes continue to vary importantly across health facilities, and many aspects of renal replacement therapy must yet be validated by high-quality evidence derived from randomized trials (22). A fundamental gap in the knowledge of renal replacement therapy is that the optimal timing for its initiation remains uncertain (22, 23). It has been hypothesized that early initiation of renal replacement therapy could be beneficial to patients because of its ability to promote better fluid balance, restore acid-base homeostasis, and remove circulating toxins accumulated due to acute kidney injury. However, the few multicenter randomized controlled trials designed to test this hypothesis have not demonstrated that early renal replacement therapy is superior to late renal replacement therapy in improving survival (23, 38).

In view of the difficulty to weight the risks and benefits of renal replacement therapy, the newly proposed quadruple therapy (39, 40) with angiotensin receptor neprilysin inhibitors (ARNI), β-blockers, mineralocorticoid receptor antagonists (MRA), and sodium-glucose cotransporter-2 (SGLT-2) inhibitors for the treatment of heart failure and reduced ejection fraction (HFrEF) may provide a long-term solution to significantly lower the rate of heart failure hospitalization and mortality in this population (41, 42).

The results of our study should be interpreted bearing its limitations in mind. Patients were identified according to clinician-judged ICD-10 codes encompassing heart failure, which due to the heterogeneity of the disease and difficulty in establishing the correct diagnosis is likely to contain a certain degree of misclassification. However, the reliance on ICD coding for diagnosis is a limitation inherent to all studies that rely on large healthcare databases of real-world information and does not prevent relevant findings from being drawn from these databases. Because the SIHSUS registry does not require the reporting of baseline comorbidities, the prevalence of many comorbidities of interest that most likely influence the outcome in heart failure, such as hypertension, diabetes, dyslipidemia, and chronic kidney disease, are likely to be underreported and therefore were prevented from being included in our multivariable analysis. In addition, the registry has no information of renal replacement therapy over time and no data regarding drug therapy, laboratory findings, previous and in-hospital cardiovascular events (i.e., non-fatal myocardial infarction, ventricular arrhythmias, cardiovascular death), and cardiac phenotype. Due to a limitation in the SIHSUS registry, it was not possible to identify the number of times a single patient was hospitalized for heart failure, and consecutive hospitalizations from the same patient were required to be handled separately. We were also prevented from identifying patients receiving renal replacement therapy prior to hospital admission and were therefore compelled to analyze these patients along with patients started on renal replacement therapy during their hospital stay. Additionally, it is important to highlight the further limitations imposed by the database utilized, which impeded a comprehensive analysis of both the specific etiology of renal replacement therapy and the background medical therapy. Finally, in spite of adjusting for a large number of confounders, residual confounding may persist due to unmeasured factors that could not be adjusted for.

## 5. Conclusion

By analyzing the records of 910,128 hospitalizations that occurred due to heart failure in the Brazilian National Public Health System (SUS) between April 2017 and August 2021, we report a worrying in-hospital all-cause mortality rate of 11.7%, surpassing by twice as much the estimates in European and North American countries. Renal replacement therapy, required by 2.2% of patients, was strongly associated with a prolonged hospital stay, higher cost-of-hospitalization, and in-hospital death. Considering the uncertain nature of renal replacement therapy's true efficacy, our findings highlight the significance of optimizing medical therapy for patients admitted with heart failure to prevent the deterioration of their renal function and the need for renal replacement therapy.

## Data Availability

The original contributions presented in the study are included in the article/**Supplementary Material**, further inquiries can be directed to the corresponding authors.

## Supplementary material

SUPPLEMENTAL FIGURE S1

Kaplan-Meier survival curve for patients stratified by pandemic status, with the pre-pandemic period represented in red and the pandemic period represented in blue, showing that patients admitted after the pandemic began tended to have worse in-hospital survival (*p* < 0.0001).

SUPPLEMENTAL FIGURE S2

The association of (**A**) income level and (**B**) longevity indices by state with in-hospital death of patients admitted with heart-failure symptoms was analyzed with a mixed effects Cox proportional hazards model, with random intercept term for each federal state. Longevity and income-level indices with a range from 0 to 1 averaged 0.83 ± 0.043, and 0.71 ± 0.053, respectively, for Brazil. Better longevity reduced the risk of death (HR = 0.80 for 0.1 change; *p* < 0.001), while higher income increased the risk (HR = 1.31 for 0.1 change of index; *p* < 0.001). Predicting these effects by federal state showed significant regional disparities: (**A**) higher incomes in the southern states of Brazil were associated with a higher risk of in-hospital death, while lower income levels in the north reduced the risk of in-hospital death. (**B**) Better longevity in the south reduced the risk of death compared to the northern states with worse longevity indices.

SUPPLEMENTAL TABLE S1

Patient characteristics stratified by renal replacement therapy.
