# Supplementary material for: Prognostic role of renal replacement therapy among hospitalized patients with heart failure in the Brazilian national public health system
Source: Front Cardiovasc Med. 2023 Aug 23;10:1226481. doi: 10.3389/fcvm.2023.1226481 (PMC10482263; doi:10.3389/fcvm.2023.1226481)
Supplement: Supplementary file 1 [file Table1.docx]

**Supplemental Table 1 (online-only Data Supplement):** Patient Characteristics Stratified By Renal Replacement Therapy

| **Supplemental Table 1:** Patient Characteristics Stratified By Renal Replacement Therapy (RRT) | | | |
| --- | --- | --- | --- |
| **Variable** | **No RRT**, N = 890,453*^1^* | **RRT**, N = 19,675*^1^* | ***p*-value***^2^* |
| Age at admission | 69 (59, 79) | 65 (56, 74) | <0.001 |
| Female sex | 430,518 (48%) | 8,459 (43%) | <0.001 |
| Race |  |  | <0.001 |
| White | 338,428 (49%) | 7,068 (44%) |  |
| Pardo | 290,259 (42%) | 7,455 (47%) |  |
| Black | 44,302 (6.4%) | 1,173 (7.3%) |  |
| Asian | 18,960 (2.7%) | 285 (1.8%) |  |
| Indigenous | 856 (0.1%) | 5 (<0.1%) |  |
| Days of hospitalization | 5.0 (3.0, 9.0) | 14 (8.0, 26) | <0.001 |
| In-hospital death | 98,204 (11%) | 8,179 (42%) | <0.001 |
| Hx diabetes | 16,600 (1.9%) | 655 (3.3%) | <0.001 |
| Hx hypertension | 38,579 (4.3%) | 1,295 (6.6%) | <0.001 |
| Hx dyslipidemia | 343 (<0.1%) | 8 (<0.1%) | 0.880 |
| Hx atrial fibrillation/flutter | 6,001 (0.7%) | 152 (0.8%) | 0.095 |
| Hx stroke | 2,420 (0.3%) | 107 (0.5%) | <0.001 |
| Hx renal insufficiency | 10,435 (1.2%) | 4,160 (21%) | <0.001 |
| RRT type |  |  | <0.001 |
| Continuous dialysis | 0 (0%) | 2,669 (14%) |  |
| Peritoneal dialysis | 0 (0%) | 315 (1.6%) |  |
| Intermittent dialysis | 0 (0%) | 16,659 (85%) |  |
| Obstructive pulmonary disease | 8,680 (1.0%) | 169 (0.9%) | 0.101 |
| Chagas' disease | 1,041 (0.1%) | 41 (0.2%) | <0.001 |
| COVID-19 infection | 360 (<0.1%) | 25 (0.1%) | <0.001 |
| Hx coronary bypass | 9 (<0.1%) | 1 (<0.1%) | 0.196 |
| In-hospital heart Tx | 670 (<0.1%) | 219 (1.1%) | <0.001 |
| Total hospitalization cost | 777 (715, 1,195) | 5,364 (2,847, 10,283) | <0.001 |
| Hospitalization cost per day | 196 (116, 354) | 441 (230, 735) | <0.001 |
| Year of hospital admission |  |  | <0.001 |
| 2017 | 205,451 (23%) | 3,711 (19%) |  |
| 2018 | 197,076 (22%) | 3,964 (20%) |  |
| 2019 | 195,542 (22%) | 4,316 (22%) |  |
| 2020 | 162,262 (18%) | 4,051 (21%) |  |
| 2021 | 130,122 (15%) | 3,633 (18%) |  |
| Geographic location of hospital |  |  | <0.001 |
| Central-West | 61,830 (6.9%) | 1,772 (9.0%) |  |
| Northeast | 200,022 (22%) | 4,430 (23%) |  |
| North | 47,876 (5.4%) | 1,226 (6.2%) |  |
| Southeast | 369,632 (42%) | 8,726 (44%) |  |
| South | 211,091 (24%) | 3,521 (18%) |  |
| *^1^* Median (IQR); n (%) | | | |
| *^2^* Kruskal-Wallis rank sum test; Pearson’s Chi-squared test; Fisher’s exact test | | | |
