# Supplementary material for: Prognostic role of renal replacement therapy among hospitalized patients with heart failure in the Brazilian national public health system
Source: Front Cardiovasc Med. 2023 Aug 23;10:1226481. doi: 10.3389/fcvm.2023.1226481 (PMC10482263; doi:10.3389/fcvm.2023.1226481)
Supplement: Supplementary file 2 [file Image1.pdf]

Supplemental Figure 1 (online-only Data Supplement)

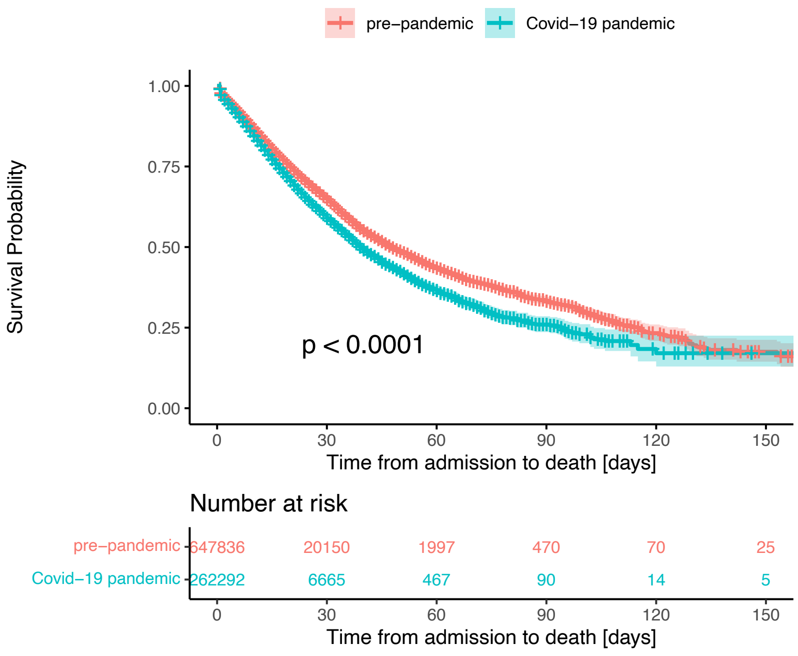

Supplemental Figure 1: Kaplan-Meier survival curve for patients stratified by pandemic status, with the pre-pandemic period represented in red and the pandemic period represented in blue, showing that patients admitted after the pandemic began tended to have worse in-hospital survival ($p < 0.001$).
