# Supplementary material for: Prognostic role of renal replacement therapy among hospitalized patients with heart failure in the Brazilian national public health system
Source: Front Cardiovasc Med. 2023 Aug 23;10:1226481. doi: 10.3389/fcvm.2023.1226481 (PMC10482263; doi:10.3389/fcvm.2023.1226481)
Supplement: Supplementary file 3 [file Image2.pdf]

Supplemental Figure 2 (online-only Data Supplement)

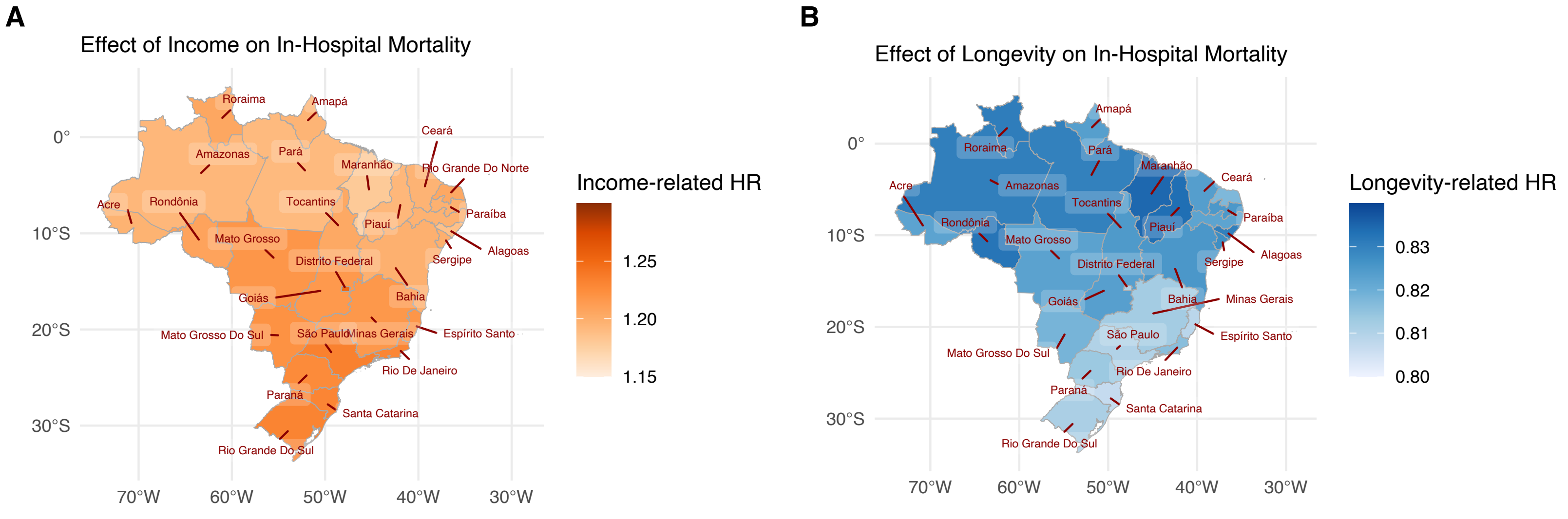

Supplemental Figure 2: The association of A) income level and B) longevity indices by state with in-hospital death of patients admitted with heart-failure symptoms was analyzed with a mixed effects Cox proportional hazards model, with random intercept term for each federal state. Longevity and income-level indices with a range from 0 to 1 averaged 0.83±0.043, and 0.71±0.053, respectively, for Brazil. Better longevity reduced the risk of death (HR 0.80 for 0.1 change; $p < 0.001$), while higher income increased the risk (HR 1.31 for 0.1 change of index; $p < 0.001$). Predicting these effects by federal state showed significant regional disparities: A) higher incomes in the southern states of Brazil were associated with a higher risk of in-hospital death, while lower income levels in the north reduced the risk of in-hospital death. B) Better longevity in the south reduced the risk of death compared to the northern states with worse longevity indices.
